# Fibrinogen Alpha Chain (FGA) Associates With Epithelial–Mesenchymal Transition, Stemness, and the USP–Catenin Complex in Colorectal Cancer

**DOI:** 10.1155/sci/9599574

**Published:** 2026-10-19

**Authors:** Hao Lin, Weixu Chen, Qing Li

**Affiliations:** ^1^ Department of Gastrointestinal Surgery Xuzhou Rehabilitation Hospital Xuzhou 221003 Jiangsu China; ^2^ Department of Gastroenterology Xuzhou Central Hospital Southeast University Xuzhou 221009 Jiangsu China seu.edu.cn; ^3^ Department of Clinical Laboratory Xuzhou Central Hospital Southeast University Xuzhou 221009 Jiangsu China seu.edu.cn

**Keywords:** cancer stem cells, colorectal cancer, epithelial–mesenchymal transition, fibrinogen alpha chain, ubiquitin-specific proteases

## Abstract

**Background:**

Colorectal cancer (CRC) progression is heavily driven by epithelial–mesenchymal transition (EMT) and the acquisition of cancer stem cell (CSC) properties. Elucidating the specific molecular drivers and dynamic indicators underlying these processes is crucial for advancing CRC management.

**Methods:**

The clinical significance and diagnostic value of fibrinogen alpha chain (FGA) were evaluated using TCGA data, immunohistochemistry (IHC), and paired pre‐ and postoperative serum samples. The biological functions of FGA were assessed in HCT116 cells through Transwell and 3D spheroid formation assays. Immunoprecipitation‐mass spectrometry (IP‐MS) and protein–protein interaction (PPI) network analyses were utilized to uncover the underlying molecular mechanisms.

**Results:**

FGA mRNA and protein are significantly upregulated in CRC tissues, and preliminary data indicate elevated serum FGA in CRC patients. Clinically, aberrant FGA expression correlates with tumor histological type, lymphatic invasion, and poor survival outcomes. In vitro, FGA overexpression is associated with the downregulation of E‐cadherin, upregulation of mesenchymal markers (Vimentin, ZEB1, Snail), and increased cell migration and spheroid formation. Mechanistically, IP‐MS identified a potential PPI network wherein FGA associates with ubiquitin‐specific proteases (USP7, USP10) and the catenin complex (β‐catenin, p120‐catenin), suggesting a potential role in mediating pro‐stemness signaling.

**Conclusion:**

These findings suggest that FGA may contribute to CRC plasticity by modulating EMT and stem‐like properties in vitro. Furthermore, its dynamic postoperative reduction warrants further investigation to determine its utility for non‐invasive tumor monitoring.

## 1. Introduction

Colorectal cancer (CRC) remains one of the most prevalent and lethal malignancies worldwide [1, 2]. The primary causes of CRC‐related mortality are tumor metastasis and disease recurrence, processes fundamentally driven by epithelial–mesenchymal transition (EMT) and the acquisition of cancer stem cell (CSC) traits [3, 4]. CSCs represent a minor subpopulation within the tumor but are highly tumorigenic, chemoresistant, and serve as the “seeds” for distant organ metastasis [5, 6]. The dynamic regulation of this stemness involves complex intracellular signaling networks, most prominently the Wnt/β‐catenin pathway, which is aberrantly activated in the vast majority of CRCs [7–9]. The stability of core regulators in these pathways, such as β‐catenin, is tightly controlled by post‐translational modifications [10, 11]. Ubiquitination typically targets these factors for proteasomal degradation, whereas deubiquitination, mediated by ubiquitin‐specific proteases (USPs), rescues them, thereby maintaining malignant cellular plasticity and promoting tumor progression [12, 13].

Fibrinogen alpha chain (FGA) is classically recognized for its role as a structural protein in the coagulation cascade, assembling with beta and gamma chains to form the fibrin matrix essential for hemostasis [14, 15]. However, emerging evidence suggests a deep interplay between the coagulation system and malignancy [16, 17]. Coagulation factors can extravasate into the tumor microenvironment (TME) and exhibit pleiotropic effects, participating in cellular signaling, extracellular matrix remodeling, and tumor cell survival [18, 19]. Indeed, elevated systemic fibrinogen is frequently observed in cancer patients and is associated with poor survival outcomes in digestive system tumors, including CRC [20, 21]. While the elevation of total serum or plasma fibrinogen in CRC due to cancer‐associated hypercoagulability and acute‐phase responses is well documented, the specific dynamic monitoring value of the FGA subunit as a blood‐based biomarker and its intracellular molecular interactome regulating EMT and stemness remain largely unexplored [22, 23]. Given the pressing need for reliable, non‐invasive diagnostic tools and a better understanding of metastatic drivers, investigating the functional role of FGA is of high clinical relevance [24, 25].

Recently, the landscape of CRC liquid biopsy has expanded significantly beyond circulating tumor DNA (ctDNA) to include advanced circulating protein and extracellular vesicle (EV)‐based biomarker discovery. High‐throughput proteomic strategies, particularly data‐independent acquisition mass spectrometry (DIA‐MS), have enabled the comprehensive screening of serum and plasma, uncovering multiplexed protein signatures that reflect early tumor dynamics and microenvironmental alterations [26, 27]. Concurrently, tumor‐derived EVs, which encapsulate rich proteomic and transcriptomic cargo, have emerged as highly stable, protected reservoirs for non‐invasive biomarker detection [28, 29]. While these high‐throughput screening and EV‐based platforms offer powerful tools for broad diagnostic signature discovery, there remains a critical need to isolate and biologically validate specific, functionally relevant protein hubs. Validating these individual targets is essential not only for developing accessible, targeted serum assays but also for understanding the underlying mechanisms that drive tumor progression.

In this study, we investigated the dual clinical and biological roles of FGA in CRC. We demonstrate that beyond its recognized role in the coagulation cascade, FGA appears to function as a modulator of EMT and cancer stemness. Additionally, we evaluate its potential for dynamic postoperative monitoring using the patient serum. Furthermore, we identify a potential protein–protein interaction (PPI) network wherein FGA exhibits putative interactions with USP deubiquitinases and the catenin complex to potentially influence pro‐stemness signaling in CRC cells.

## 2. Materials and Methods

### 2.1. Clinical Data and Bioinformatics Analysis

Gene expression profiles and clinicopathological data of 644 CRC patients were obtained from the TCGA database. Survival analysis was performed using LASSO Cox regression to evaluate overall survival (OS), disease‐specific survival (DSS), and progression‐free Interval (PFI). Additionally, a large‐scale external validation cohort comprising 1061 CRC patients from independent GEO microarray datasets was analyzed using the Kaplan–Meier Plotter database (http://www.kmplot.com/analysis/).

### 2.2. Tissue and Serum Assays

FGA protein levels in paired normal and CRC tumor tissues were evaluated using immunohistochemistry (IHC). Serum FGA expression levels were assessed in samples from 71 CRC patients (both pre‐ and postoperatively, collected 7 days post‐resection) and 47 healthy controls. The collection and use of human clinical samples were approved by the Institutional Ethics Committee of Xuzhou Central Hospital (Approval No. XK‐LW‐20230529‐014).

To specifically quantify the target protein rather than total functional fibrinogen, serum FGA concentrations were measured using a commercially available human fibrinogen alpha chain precursor isoform 1 (FGA) ELISA Kit (Vicmed, China). This assay utilizes monoclonal antibodies highly specific to the FGA subunit, distinguishing it from conventional clinical plasma fibrinogen testing, which relies on thrombin‐induced clotting of the intact fibrinogen hexamer in citrated plasma. For sample preparation, peripheral blood was collected in standard serum separator tubes, allowed to clot at room temperature for 30 min, and centrifuged at 1000 × *g* for 15 min. The isolated serum was immediately aliquoted and stored at −80°C. All samples were analyzed, avoiding repeated freeze–thaw cycles (maximum one cycle). The assays were performed strictly according to the manufacturer’s protocol, utilizing a standard calibration curve. Quality control was ensured by assessing intra‐assay and inter‐assay coefficients of variation (CV), which were <8% and <10%, respectively. Diagnostic efficacy was determined via receiver operating characteristic (ROC) curve analysis.

### 2.3. Cell Culture and Transfection

The CRC cell line HCT116 was obtained from the American Type Culture Collection (ATCC). Cells were cultured in DMEM/high glucose (VCM12800PS, Vicmed, China) supplemented with 10% fetal bovine serum (FBS) and maintained in a humidified incubator at 37°C with 5% CO_2_. The cells were transfected with flag‐tagged FGA plasmids (FlagFGA) or control plasmids (FlagCtrl) to establish an overexpression model. For loss‐of‐function experiments, cells were transfected with specific short hairpin RNAs (shRNAs) targeting FGA (shFGA) or negative control shRNAs (shNC).

### 2.4. In Vitro Functional Assays

EMT marker expression (E‐cadherin, Vimentin, ZEB1, and Snail) and cancer stemness markers (OCT4 and SALL4) were analyzed via Western blot. Cell migratory capacity was assessed using Transwell assays. Tumor stemness was evaluated utilizing a 3D spheroid formation assay.

### 2.5. Immunoprecipitation‐Mass Spectrometry (IP‐MS)

FGA–interacting protein complexes were isolated via immunoprecipitation and analyzed using tandem mass spectrometry (MS/MS). Enrichment profiles were generated by evaluating the Sum PEP Scores and unique peptide counts.

### 2.6. PPI Network Construction

The molecular network orchestrating FGA functions was visualized by integrating the IP‐MS dataset with known protein interactions retrieved from the STRING public database.

### 2.7. Reciprocal Co‐Immunoprecipitation (Co‐IP) and Ubiquitination Assays

To validate protein interactions, cells were lysed in RIPA buffer supplemented with protease and phosphatase inhibitors. Reciprocal Co‐IP was performed using specific antibodies against FGA, USP10, and β‐catenin, followed by incubation with protein A/G magnetic beads. The immunoprecipitated complexes were analyzed by Western blotting. For protein stability assays, cells were treated with the proteasome inhibitor MG132 (10 μM) for a time course ranging from 0 to 14 h. Ubiquitination levels were assessed by immunoprecipitating the target protein and immunoblotting with an anti‐ubiquitin antibody following MG132 treatment.

### 2.8. Statistical Analysis

All statistical analyses were performed using R software (version 4.2.1) and GraphPad Prism (version 9.0). Continuous variables were compared using Student’s *t*‐test or the Mann–Whitney *U* test as appropriate. Categorical variables were analyzed using the Chi‐square test. A *p*‐value of <0.05 was considered statistically significant.

## 3. Results

### 3.1. Upregulated Expression and Clinical Significance of FGA in CRC

To investigate the role of FGA in CRC, we first analyzed its mRNA expression levels in CRC tissues and adjacent normal tissues. The results demonstrated that FGA expression was significantly upregulated in CRC tumor tissues compared to normal tissues in both paired (Figure 1A) and unpaired (Figure 1B) sample analyses (*p* < 0.001). Consistent with the transcriptional data, IHC staining further confirmed across nine distinct cases that FGA protein levels were markedly elevated in CRC tissues relative to paired normal tissues (Figure 1G).

**Figure 1:**
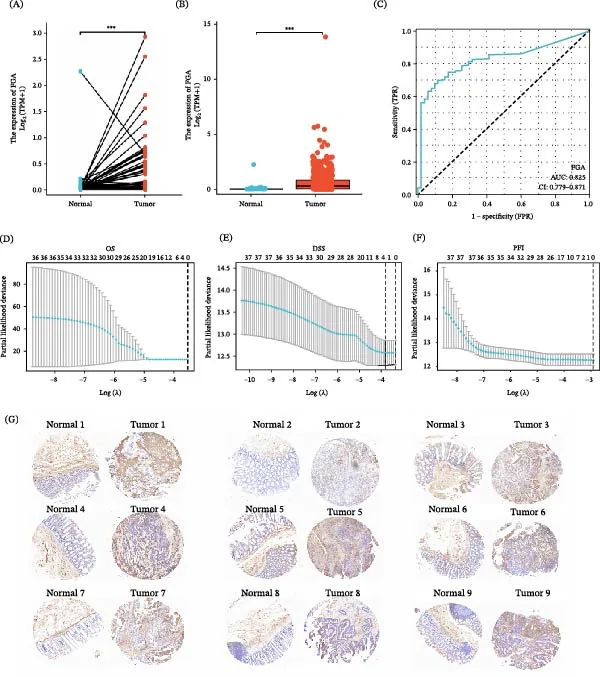
Expression profile, diagnostic value, and prognostic significance of FGA in colorectal cancer (CRC). (A) Paired analysis of FGA mRNA expression (log_2_(TPM + 1)) between CRC tumor tissues and adjacent normal tissues (*n* = 51 paired cases). (B) Unpaired expression analysis of FGA (log_2_(TPM + 1) in normal (*n* = 51) and CRC tumor (*n* = 644) tissues. Data are presented as median with interquartile range.  ^∗∗∗^ *p* < 0.001. (C) Receiver operating characteristic (ROC) curve analysis evaluating the diagnostic performance of FGA in distinguishing CRC from normal tissues (AUC = 0.825, 95% CI: 0.779–0.871). (D–F) Least absolute shrinkage and selection operator (LASSO) Cox regression analysis showing the partial likelihood deviance versus log(*λ*) for (D) overall survival (OS), (E) disease‐specific survival (DSS) and (F) progression‐free interval (PFI). The dashed vertical lines indicate the optimal *λ* values determined by cross‐validation. (G) Representative IHC images demonstrating FGA protein expression levels in paired normal and CRC tumor tissues across 9 distinct cases. Statistical significance for continuous variables was determined using the Mann–Whitney *U* test for unpaired data and the Wilcoxon matched‐pairs signed‐rank test for paired data.

Next, we evaluated the diagnostic and prognostic value of FGA in CRC. ROC curve analysis revealed that FGA exhibited a strong diagnostic ability to distinguish tumors from normal tissues, with an area under the curve (AUC) of 0.825 (95% CI: 0.779–0.871) (Figure 1C). Furthermore, LASSO Cox regression analysis was performed to assess the prognostic significance of FGA. The partial likelihood deviance plots indicated the robust prognostic value of the signature involving FGA across multiple survival outcomes, including OS (Figure 1D), DSS (Figure 1E), and PFI (Figure 1F). Together, these findings strongly suggest that FGA is overexpressed in CRC and may serve as a potential diagnostic and prognostic biomarker.

### 3.2. Elevated Serum FGA Levels and Its Diagnostic Value in CRC

To evaluate the potential of FGA as a circulating non‐invasive biomarker for CRC, we assessed the relative expression levels of FGA in serum samples from 71 CRC patients and 47 healthy controls. The results demonstrated that serum FGA levels were significantly elevated in the CRC group compared to the normal control group (Figure 2A, *p* < 0.001). To further explore the relationship between FGA levels and tumor burden, we tracked the dynamic changes in serum FGA before and after surgical tumor resection. Paired sample analysis revealed a significant decrease in circulating FGA levels postoperatively compared to the preoperative baseline (Figure 2B, *p* < 0.01). While this dynamic reduction is an interesting observation, postoperative FGA levels can be heavily influenced by confounding perioperative factors, specifically surgery‐related inflammation, activation of the coagulation cascade, fluid administration, and transient alterations in liver function. Therefore, these data suggest a potential link between the tumor burden and circulating FGA rather than definitively proving the primary tumor as the sole source.

**Figure 2:**
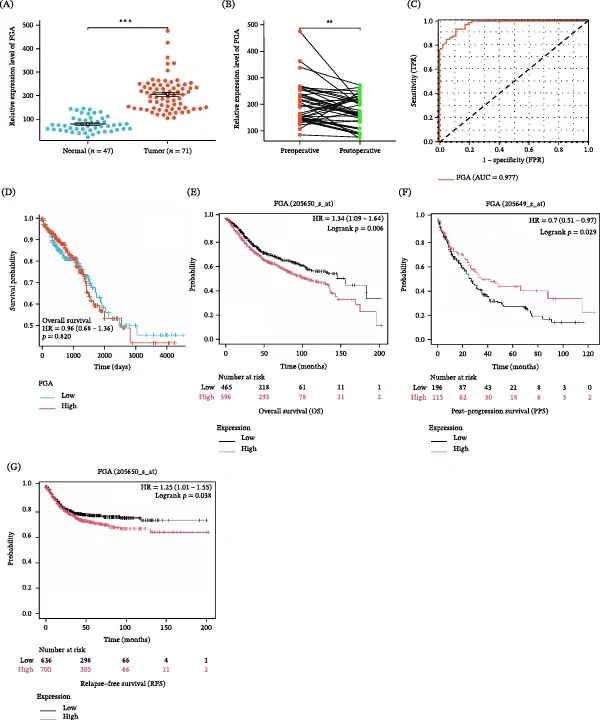
Serum FGA expression, diagnostic efficacy, and external prognostic validation in colorectal cancer. (A) Scatter plot showing the relative expression levels of FGA in serum samples from normal healthy controls (*n* = 47) and CRC patients (tumor, *n* = 71).  ^∗∗∗^ *p* < 0.001, determined by the Mann‐Whitney *U* test. (B) Paired analysis of relative FGA expression levels in the serum of CRC patients before (preoperative) and 7 days after (postoperative) surgical tumor resection (*n* = 71). *p* < 0.01, determined by the Wilcoxon matched‐pairs signed‐rank test. (C) Receiver operating characteristic (ROC) curve analysis evaluating the diagnostic performance of serum FGA in distinguishing CRC patients from healthy controls (AUC = 0.977). (D) Kaplan–Meier survival curve for overall survival (OS) based on bulk FGA mRNA expression in the primary TCGA CRC cohort (HR = 0.96, 95% CI: 0.68–1.36, *p* = 0.820). (E–G) External validation of FGA using large‐scale, independent GEO microarray cohorts (*n* = 1061) via the Kaplan–Meier Plotter database. Kaplan–Meier survival curves demonstrate (E) OS (HR = 1.34, 95% CI: 1.09–1.64, *p* = 0.006), (F) post‐progression survival (PPS) (HR = 0.70, 95% CI: 0.51–0.97, *p* = 0.029), and (G) relapse‐free survival (RFS) (HR = 1.25, 95% CI: 1.01–1.55, *p* = 0.038). Survival differences in (D–G) were evaluated using the log‐rank test.

Furthermore, a ROC curve analysis was performed to evaluate the diagnostic efficacy of serum FGA in differentiating CRC patients from healthy individuals. The preliminary analysis yielded an AUC of 0.977 in this specific cohort (Figure 2C). However, it is crucial to emphasize that this initial analysis was based exclusively on comparing CRC patients to healthy controls and did not include clinically relevant disease controls, such as patients with benign colorectal adenomas or inflammatory bowel disease (IBD). Given FGA’s known association with systemic inflammation and coagulation, its true disease specificity against other colorectal pathologies cannot be established from this cohort. Nevertheless, coupled with the observed postoperative dynamics, these preliminary data suggest that monitoring circulating FGA may have potential utility in evaluating the tumor burden, warranting further validation in larger, clinically diverse cohorts.

### 3.3. Association Between FGA Expression and Clinicopathological Characteristics in CRC

To further evaluate the clinical significance of FGA in CRC, we investigated the association between FGA expression levels and various clinicopathological features using clinical data from the TCGA database (Table 1). A total of 644 CRC patients were divided into low‐expression (*n* = 322) and high‐expression (*n* = 322) groups.

**Table 1:** The relationship between FGA expression levels and various clinicopathological features.

Characteristics	Low expression of FGA	High expression of FGA	*p*‐Value
*n*	322	322	—
Gender, *n* (%)			0.385
Female	156 (24.2%)	145 (22.5%)	—
Male	166 (25.8%)	177 (27.5%)	—
Age, *n* (%)			0.873
≤65	139 (21.6%)	137 (21.3%)	—
>65	183 (28.4%)	185 (28.7%)	—
BMI, *n* (%)			0.385
≤25	58 (17.6%)	49 (14.9%)	—
>25	109 (33.1%)	113 (34.3%)	—
Pathologic T stage, *n* (%)			0.258
T1 and T2	59 (9.2%)	72 (11.2%)	—
T3	221 (34.5%)	215 (33.5%)	—
T4	42 (6.6%)	32 (5%)	—
Pathologic N stage, *n* (%)			0.455
N0	180 (28.1%)	188 (29.4%)	—
N1	76 (11.9%)	77 (12%)	—
N2	66 (10.3%)	53 (8.3%)	—
Pathologic M stage, *n* (%)			0.261
M0	239 (42.4%)	236 (41.8%)	—
M1	39 (6.9%)	50 (8.9%)	—
Pathologic stage *n* (%)			0.096
Stage I	50 (8%)	61 (9.8%)	—
Stage II	119 (19.1%)	119 (19.1%)	—
Stage III	105 (16.9%)	79 (12.7%)	—
Stage IV	39 (6.3%)	51 (8.2%)	—
Primary therapy outcome, *n* (%)			0.798
PD	17 (5.4%)	16 (5.1%)	—
SD	3 (1%)	2 (0.6%)	—
PR	9 (2.9%)	7 (2.2%)	—
CR	121 (38.8%)	137 (43.9%)	—
Histological type, *n* (%)			< 0.001
Adenocarcinoma	257 (40.6%)	293 (46.3%)	—
Mucinous adenocarcinoma	60 (9.5%)	23 (3.6%)	—
Residual tumor, *n* (%)			0.232
R0	242 (47.5%)	226 (44.3%)	—
R1	2 (0.4%)	4 (0.8%)	—
R2	14 (2.7%)	22 (4.3%)	—
CEA level, *n* (%)			0.975
≤5	136 (32.8%)	125 (30.1%)	—
>5	80 (19.3%)	74 (17.8%)	—
Perineural invasion, *n* (%)			0.080
No	85 (36.2%)	90 (38.3%)	—
Yes	37 (15.7%)	23 (9.8%)	—
Lymphatic invasion, *n* (%)			0.030
No	164 (28.2%)	186 (32%)	—
Yes	130 (22.3%)	102 (17.5%)	—
History of colon polyps, *n* (%)			0.241
No	179 (32.3%)	198 (35.7%)	—
Yes	94 (16.9%)	84 (15.1%)	—
Colon polyps present, *n* (%)			0.172
No	106 (32.8%)	118 (36.5%)	—
Yes	55 (17%)	44 (13.6%)	—

*Note*: The sum of patients in certain subgroups (such as pathologic stage, residual tumor, and so on) does not equal the total number of patients (*n* = 322 per group) due to missing or unknown clinical data within the TCGA database.

Statistical analysis revealed that FGA expression was significantly associated with the histological type of the tumor (*p* < 0.001). Specifically, patients in the high FGA expression group had a higher proportion of standard adenocarcinoma (46.3% vs. 40.6%), whereas mucinous adenocarcinoma was notably more frequent in the low FGA expression group (9.5% vs. 3.6%). Furthermore, FGA expression showed a significant inverse correlation with lymphatic invasion (*p* = 0.030). Specifically, the incidence of lymphatic invasion was higher in the low FGA expression cohort (22.3%) compared to the high expression cohort (17.5%).

There were no statistically significant correlations observed between FGA expression and other baseline demographic or clinical characteristics, including gender (*p* = 0.385), age (*p* = 0.873), body mass index (BMI) (*p* = 0.385), preoperative CEA levels (*p* = 0.975), primary therapy outcome (*p* = 0.798), or TNM staging (Pathologic T, N, and M stages; all *p* > 0.05) (Table 1). Marginal trends without statistical significance were noted for pathologic stage (*p* = 0.096) and perineural invasion (*p* = 0.080).

In summary, the clinicopathological analysis indicates that aberrant FGA expression is closely associated with specific intrinsic tumor characteristics, namely, histological type and lymphatic invasion status, rather than general demographic factors, conventional TNM staging, or preoperative CEA levels. These findings suggest that FGA represents an independent molecular feature that reflects distinct biological behaviors in CRC, highlighting its potential utility as a specialized biomarker complementary to existing clinical evaluation systems.

To rigorously quantify the prognostic value of FGA and clarify its independence from standard clinical metrics, we performed Kaplan–Meier survival analysis and Cox proportional hazards regression analyses within the TCGA CRC cohort (Figure 2D, Table 2). Following adjustment for standard clinicopathological variables, including TNM stage, CEA levels, and lymphatic invasion, bulk FGA mRNA expression in primary tumors did not reach statistical significance as an independent prognostic factor for OS in this specific cohort (multivariate HR = 1.44, 95% CI: 0.66–3.15, *p* = 0.357). This aligns with the clinicopathological characteristics observed in Table 1, suggesting that static FGA expression in the primary tumor bulk exhibits complex, nonlinear dynamics during early local invasion.

**Table 2:** Univariate and multivariate Cox regression analysis of FGA expression and clinicopathological variables for overall survival in the TCGA CRC cohort.

Clinicopathological variables	Univariate analysis HR (95% CI)	*p*‐Value	Multivariate analysis HR (95% CI)	*p*‐Value
FGA expression (high vs. low)	1.49 (0.90–2.49)	0.123	1.44 (0.66–3.15)	0.357
Age (>65 vs. ≤65)	2.11 (1.20–3.70)	**0.009**	2.21 (0.96–5.09)	0.061
Gender (male vs. female)	1.42 (0.85–2.37)	0.186	0.82 (0.38–1.77)	0.611
Pathologic stage (III–IV vs. I–II)	1.87 (1.11–3.15)	**0.018**	1.15 (0.41–3.19)	0.789
CEA level (>5 vs. ≤5)	3.33 (1.53–7.25)	**0.002**	2.73 (1.20–6.20)	**0.017**
Lymphatic invasion (yes vs. no)	2.12 (1.19–3.77)	**0.01**	3.45 (1.24–9.62)	**0.018**

*Note*: Statistically significant *p*‐values (*p* < 0.05) are highlighted in bold. The multivariate Cox proportional hazards regression model was adjusted for the standard clinicopathological variables listed in the table to evaluate independent prognostic significance.

Abbreviations: CEA, carcinoembryonic antigen; CI, confidence interval; CRC, colorectal cancer; HR, hazard ratio; OS, overall survival; TCGA, The Cancer Genome Atlas.

Consequently, rigorous independent external validation is requisite to determine systemic clinical applicability. To achieve this, we performed extensive survival analysis using large‐scale external validation cohorts via the Kaplan–Meier Plotter database (*n* = 1061), integrating independent GEO microarray datasets. This robust external validation conclusively demonstrated that CRC patients with high FGA expression exhibited significantly poorer OS compared to the low‐expression cohort (HR = 1.34, 95% CI: 1.09–1.64, *p* = 0.006; Figure 2E). Furthermore, elevated FGA expression was significantly associated with worse post‐progression survival (PPS) (HR = 0.70, 95% CI: 0.51–0.97, *p* = 0.029; Figure 2F) and relapse‐free survival (RFS) (HR = 1.25, 95% CI: 1.01–1.55, *p* = 0.038; Figure 2G). These external validations suggest that elevated FGA expression is associated with poorer survival outcomes, highlighting its potential as a prognostic indicator in CRC.

### 3.4. FGA Modulates EMT, Migration, and Stem‐Like Properties in CRC Cells

Given the complex in vivo relationship between FGA expression and lymphatic invasion, which suggests a highly dynamic role during tumor progression, we sought to directly isolate and investigate the biological functions of FGA on cell motility and plasticity in vitro. We established both FGA‐overexpressing (FlagFGA) and FGA‐knockdown (ShFGA) models in the HCT116 CRC cell line.

Because EMT is a crucial mechanism driving tumor invasion and metastasis, we first examined the expression levels of EMT‐related markers. Western blot analysis revealed that ectopic expression of FGA markedly downregulated the epithelial marker E‐cadherin while simultaneously upregulating mesenchymal markers (Vimentin, ZEB1, and Snail) and core stemness factors (SALL4 and OCT4) (Figure 3A). Conversely, FGA knockdown effectively reversed this molecular phenotype, restoring E‐cadherin expression and suppressing the mesenchymal and stemness markers (Figure 3B).

**Figure 3:**
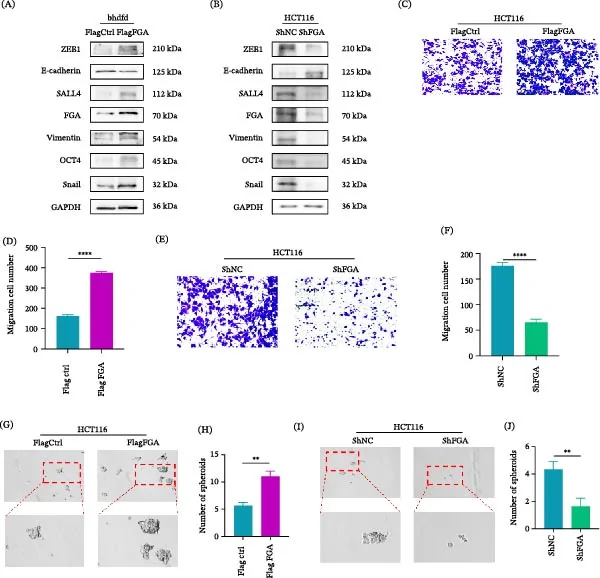
FGA modulates epithelial–mesenchymal transition (EMT), cell migration, and spheroid formation in HCT116 colorectal cancer cells. (A, B) Western blot analysis evaluating the protein expression levels of EMT‐related markers (ZEB1, E‐cadherin, Vimentin, Snail) and stemness factors (SALL4, OCT4) following (A) FGA overexpression (FlagFGA) or (B) FGA knockdown (ShFGA). GAPDH served as the internal loading control. (C–F) Transwell migration assays demonstrating the migratory ability of HCT116 cells upon (C, D) FGA overexpression and (E, F) FGA knockdown. Representative images (C, E) and quantitative analyses (D, F) are shown.  ^∗∗∗∗^ *p* < 0.0001. (G–J) Tumor spheroid formation assays evaluating stem‐like properties. Representative bright‐field images are shown for (G) FGA overexpression and (I) FGA knockdown, with red dashed boxes indicating the magnified areas displayed in the lower panels. Quantitative analyses of the number of spheroids are shown in (H, J).  ^∗∗^ *p* < 0.01. All in vitro experiments were independently repeated three times (*n* = 3 biological replicates). Data are presented as mean ± SD. Statistical significance was determined using the unpaired two‐tailed Student’s *t*‐test.

Consistent with this molecular phenotypic shift, Transwell assays demonstrated that FGA overexpression significantly enhanced the migratory capacity of HCT116 cells (Figure 3C,D), whereas FGA depletion profoundly impaired cell migration (Figure 3E,F). Furthermore, we evaluated the effect of FGA on tumor stem‐like properties using 3D spheroid formation assays. HCT116 cells overexpressing FGA formed notably larger and more numerous tumor spheroids than the control cells (Figure 3G,H). In stark contrast, FGA knockdown significantly abrogated the spheroid formation ability (Figure 3I,J). Taken together, these complementary gain‐ and loss‐of‐function data suggest that FGA modulates EMT, cell migration, and stem‐like properties in CRC cells, aligning with our exploratory hypothesis.

### 3.5. IP‐MS Profiling Identifies Putative Interactions of FGA With USP Deubiquitinases and the Catenin Complex

To explore the potential molecular mechanisms underlying the FGA‐associated EMT and enhanced stem‐like properties in HCT116 cells, we performed an IP‐MS analysis. The high‐confidence identification of peptides within the IP complex was robustly validated by tandem mass spectrometry (MS/MS), with a representative fragmentation spectrum verifying the accurate peptide sequencing shown in Figure 4A. Strikingly, functional annotation of the FGA‐interacting proteins revealed a highly specific enrichment of the catenin protein family and associated adhesion complex molecules. As visualized in the enrichment profile (Figure 4B,C), FGA exhibited strong binding affinities to crucial EMT and adhesion regulators, notably CTNND1 (p120‐catenin) and JUP (γ‐catenin/Plakoglobin), alongside the core Wnt/stemness driver CTNNB1 (β‐catenin) and STAT3. Furthermore, our IP‐MS dataset robustly captured multiple members of the USP family, particularly USP7 and USP10, which are well‐documented deubiquitinases known to stabilize β‐catenin [30, 31].

**Figure 4:**
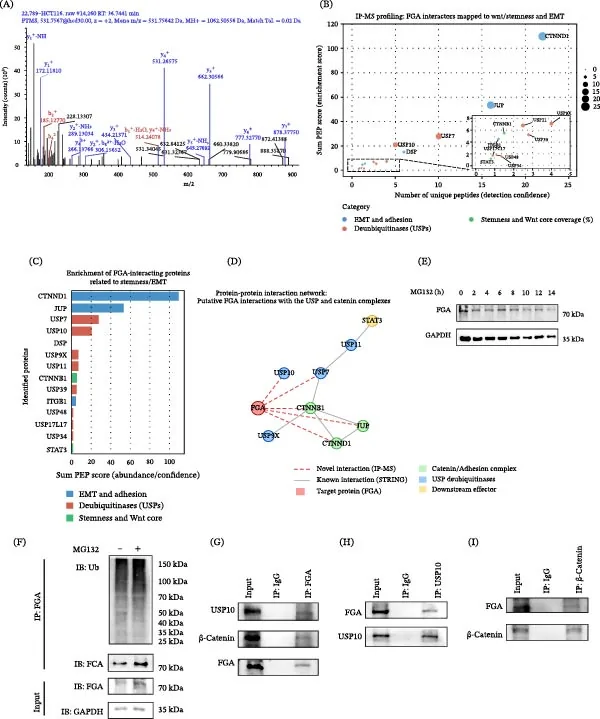
IP‐MS profiling identifies putative interactions of FGA with USP deubiquitinases and the catenin complex. (A) A representative tandem mass spectrometry (MS/MS) fragmentation spectrum identifying a unique peptide of FGA (amino acid sequence: ALTDMPQMR) from the FGA immunoprecipitate. The annotated b‐ions (red) and y‐ions (blue) confirm the accuracy of the peptide sequence match. (B) Bubble plot illustrating the enrichment of specific FGA‐interacting proteins categorized by their functions (EMT & Adhesion, USPs, and Stemness & Wnt Core). The *x*‐axis represents the number of unique peptides (detection confidence), the *y*‐axis indicates the sum PEP score (enrichment abundance), and the bubble size correlates with sequence coverage (%). (C) Bar chart ranking the top identified interacting proteins associated with stemness and EMT based on their sum PEP scores. (D) A proposed protein–protein interaction (PPI) network centered on FGA. Gray solid lines represent known interactions retrieved from the STRING database, while red dashed lines highlight the novel physical interactions identified in our IP‐MS analysis. Nodes are color‐coded according to their functional complexes. (E) Western blot analysis of FGA protein stability. HCT116 cells were treated with the proteasome inhibitor MG132 (10 μM) for the indicated times (0–14 h), showing time‐dependent FGA accumulation. GAPDH served as the loading control. (F) Ubiquitination assay of FGA. Cell lysates, with or without MG132 treatment, were immunoprecipitated (IP) with an anti‐FGA antibody and immunoblotted (IB) for ubiquitin (Ub) and FGA. (G–I) Reciprocal co‐immunoprecipitation (Co‐IP) assays validating the physical interactions among FGA, USP10, and β‐catenin. Cell lysates were immunoprecipitated with IgG (negative control) or specific antibodies against (G) FGA, (H) USP10, and (I) β‐catenin, followed by immunoblotting for the indicated target proteins. All Western blot, ubiquitination, and Co‐IP experiments were independently repeated three times (*n* = 3 biological replicates).

To further delineate the molecular network orchestrating these processes, we constructed a PPI network integrating our IP‐MS dataset with known interactions from the STRING public database (Figure 4D). Notably, querying the STRING database revealed no previously documented functional or physical associations between FGA and either the catenin/adhesion complex (CTNNB1, CTNND1, and JUP) or the USP family. Therefore, our IP‐MS data provide the first direct experimental evidence of a highly novel, previously uncharacterized interactome centered on FGA in CRC cells.

Given that FGA is primarily a structural protein lacking intrinsic catalytic activity, we hypothesize that it may participate in a multi‐protein complex regulated by the ubiquitin‐proteasome system. To investigate this functional regulation, we conducted protein stability and ubiquitination assays. Treatment of CRC cells with the proteasome inhibitor MG132 led to a marked, time‐dependent accumulation of FGA, peaking at 6 h (Figure 4E). Crucially, subsequent ubiquitination assays verified that FGA undergoes extensive polyubiquitination, which is significantly enriched upon MG132 treatment (Figure 4F). To move beyond the in silico PPI network and directly validate the physical interactions underlying this mechanism, we performed reciprocal Co‐IP assays. Immunoprecipitation of FGA robustly pulled down both USP10 and β‐catenin (Figure 4G). Reversely, immunoprecipitation of USP10 (Figure 4H) and β‐catenin (Figure 4I) successfully pulled down FGA, providing biochemical evidence for the spatial association of these proteins in CRC cells. The observed spatial association of FGA with both specific USPs and the catenin complex highlights a potential intersection between the ubiquitin–proteasome system and pro‐stemness Wnt signaling. This putative network may contribute to the stabilization of core effectors like β‐catenin, though the precise dynamics of the complex assembly require further investigation.

## 4. Discussion

The identification of reliable circulating biomarkers and the elucidation of mechanisms governing tumor stemness are critical priorities in CRC research [32]. In the present study, we investigate the potential functional role of FGA in modulating cellular plasticity in CRC and evaluate its potential utility in dynamic postoperative monitoring.

Clinically, we observed that FGA expression is significantly upregulated in CRC tissues and correlates with poor prognostic outcomes, including shortened OS, DSS, and PFI. Notably, serum FGA levels were markedly elevated in CRC patients and exhibited excellent diagnostic performance (AUC = 0.977). The significant postoperative decrease in circulating FGA highlights its potential as a dynamic, non‐invasive biomarker for monitoring the tumor status. Because FGA is acutely sensitive to perioperative factors, including surgery‐related inflammation, coagulation cascade activation, fluid administration, and liver function alterations, this postoperative reduction suggests a potential correlation with tumor removal rather than definitive proof that the primary tumor is the exclusive source of circulating FGA. Furthermore, clinicopathological analysis of TCGA data linked aberrant FGA expression to specific intrinsic tumor characteristics, specifically standard adenocarcinoma histology and an inverse correlation with lymphatic invasion, independent of conventional TNM staging or preoperative CEA levels. Notably, while FGA was not identified as an independently significant prognostic factor in the multivariable analysis of the TCGA cohort, it demonstrated a stronger prognostic value in the larger, pooled external GEO datasets (*n* = 1061). This discrepancy is likely attributable to differences in statistical power, diverse cohort compositions, and tumor heterogeneity. Bulk RNA sequencing in the TCGA cohort predominantly reflects the core of the primary tumor, which, as our clinicopathological analysis indicates, exhibits complex, nonlinear dynamics during early local invasion. In contrast, the much larger pooled GEO datasets may capture broader systemic prognostic trends. Therefore, while these external validations suggest that elevated FGA has prognostic potential, its definitive utility as an independent prognostic indicator requires cautious interpretation and further prospective validation.

The potential utility of FGA as a dynamic monitoring tool must be contextualized within the rapidly advancing field of CRC liquid biopsy. Recent breakthroughs in DIA‐MS‐based proteomics and EV isolation have shifted the paradigm toward multiplexed, high‐dimensional biomarker panels for CRC screening [33]. While EV and DIA‐MS approaches excel in broad diagnostic screening by capturing complex systemic signatures, their clinical translation is often constrained by high technical complexity and cost. Our findings regarding serum FGA complement these high‐throughput approaches by offering a focused, single‐target perspective. Rather than serving as a standalone screening tool to rival multiplexed panels, FGA tracking offers a potentially cost‐effective, easily accessible metric for dynamic postoperative monitoring. Furthermore, whereas large‐scale proteomic screens primarily provide diagnostic correlations, our study bridges the gap between liquid biopsy detection and intracellular function, delineating how this specific circulating protein actively drives EMT and stemness within the tumor.

Interestingly, while our clinical data indicate that high bulk tumor FGA expression is associated with lower lymphatic invasion, our in vitro investigations revealed that FGA overexpression in HCT116 cells significantly promotes EMT (characterized by the downregulation of E‐cadherin and upregulation of Vimentin, ZEB1, and Snail) and enhances cell migration [34]. This apparent paradox highlights the complex, stage‐dependent nature of tumor plasticity. It is well‐established that EMT is a dynamic and reversible process; while EMT is required for initial local invasion and motility, tumor cells often undergo mesenchymal‐to‐epithelial transition (MET) to successfully establish themselves in lymphatic vessels or distant metastatic niches. Additionally, FGA expression in the tumor core (as largely represented in bulk TCGA RNA‐seq data) may reflect a fundamentally different microenvironmental state than that at the invasive front. These dynamic complexities emphasize the need to understand FGA’s precise intracellular roles. Crucially, for the context of tumor progression and therapy resistance, FGA also significantly augmented cancer stem‐like properties, as evidenced by increased 3D tumor spheroid formation [35].

To define the mechanistic basis of these phenotypes, our IP‐MS analysis uncovered a highly novel FGA interactome. While public databases like STRING showed no previously documented functional associations between FGA and intracellular signaling complexes, our experimental data revealed strong binding affinities between FGA, members of the catenin family (CTNNB1, CTNND1, and JUP), and USP deubiquitinases (USP7 and USP10). Because FGA lacks intrinsic catalytic activity, we propose a putative interaction network involving FGA, USP10, and β‐catenin. By potentially associating USPs with the catenin complex, FGA facilitates the removal of polyubiquitin chains, rescuing these pro‐stemness and EMT drivers from proteasomal degradation. This stabilization of effectors like β‐catenin provides a compelling mechanistic explanation for the hyperactivation of stem‐like transcriptional programs observed in CRC cells [36, 37].

A critical conceptual consideration in our proposed interaction model is the subcellular localization of FGA. Canonically, FGA is synthesized and secreted into the plasma to function within the coagulation cascade. However, our reciprocal Co‐IP assays, performed on thoroughly washed whole‐cell lysates, demonstrate that FGA associates with USP10 and β‐catenin within the cellular environment. Importantly, we acknowledge that these reciprocal Co‐IP results demonstrate spatial protein association but do not definitively establish direct binary binding, intrinsic scaffold activity, direct β‐catenin stabilization, or subsequent Wnt pathway activation. The precise mechanism by which FGA localizes intracellularly in CRC cells remains to be fully elucidated. Tumor cells frequently exhibit dysregulated protein trafficking and secretory pathways, which can lead to the aberrant intracellular retention of otherwise secreted proteins. Alternatively, tumor‐derived FGA may undergo rapid autocrine re‐internalization, potentially via cell‐surface integrins, directing it into cytoplasmic or endosomal compartments where it intersects with the Wnt/catenin axis. We acknowledge that the lack of direct visual spatial mapping via immunofluorescence or nucleocytoplasmic fractionation is a limitation of the present study. Future investigations will be strictly required to delineate the precise subcellular coordinates (cytoplasmic versus nuclear) of the FGA–USP–catenin complex and to determine exactly how these putative interactions influence the dynamic nuclear translocation of β‐catenin.

It is important to acknowledge the limitations of our diagnostic analysis. The reported serum AUC of 0.977 should be interpreted with strict caution as our preliminary evaluation relied on a simple case–control design comparing CRC patients to healthy controls. Critically, this analysis did not include clinically relevant disease controls. This design inherently overestimates diagnostic performance in a real‐world setting because it cannot distinguish malignancy from benign inflammatory states. Given that FGA is an acute‐phase reactant intimately involved in coagulation and systemic inflammation, its diagnostic specificity may be significantly limited by confounding conditions such as benign colorectal adenomas, IBD, liver disease, or other inflammatory states. Furthermore, our current study did not benchmark FGA against established clinical screening tools, such as the fecal immunochemical test (FIT) or standard tumor markers like CEA and CA19‐9. Future prospective studies incorporating comprehensive disease‐control cohorts are essential to rigorously define the true diagnostic or monitoring viability of FGA.

Beyond the clinical evaluations, our in vitro functional conclusions regarding EMT and stem‐like properties must also be interpreted with appropriate caution. The current experimental evidence was obtained primarily using a single CRC cell line (HCT116). Furthermore, we did not perform pathway‐specific rescue experiments (such as Wnt/β‐catenin inhibition) to definitively confirm that the observed phenotypic shifts are exclusively dependent on this specific signaling axis. Consequently, while our data demonstrate an association between FGA expression and cellular plasticity, the proposed mechanism remains a candidate hypothesis that necessitates comprehensive future validation across multiple, diverse CRC cell models.

In summary, this exploratory study demonstrates that FGA is overexpressed in CRC tissues and serum. While we acknowledge its complex role as an acute‐phase reactant intimately involved in systemic inflammation, our preliminary findings suggest its potential utility for dynamic postoperative monitoring. In vitro, FGA appears to contribute to tumor plasticity by modulating EMT and stemness. Furthermore, our IP‐MS data generate a candidate hypothesis wherein FGA exhibits putative interactions with USP deubiquitinases and the catenin complex, potentially intersecting with proteasomal degradation pathways. While these findings highlight the FGA–USP–catenin axis as an interesting molecular feature of CRC, substantial prospective clinical validation and in‐depth mechanistic studies are required to confirm its true diagnostic specificity and therapeutic potential.

## Author Contributions

Qing Li designed the research. Hao Lin and Weixu Chen were responsible for data collection under the supervision of Qing Li. Hao Lin and Weixu Chen conceived and planned the experiments and analyzed the data. Hao Lin and Weixu Chen wrote the first draft of the manuscript and Qing Li critically reviewed and revised the manuscript for important intellectual content and also improved by all authors. Hao Lin, Weixu Chen, and Qing Li were responsible for the final content.

## Funding

This project is supported by the General Project of Xuzhou Basic Research Plan in 2023 (Grant KC23070), Xuzhou Municipal Health Commission 2024 Youth Medical Science and Technology Innovation Project (Grant XWKYHT20240016), and Science and Technology Development Fund of Affiliated Hospital of Xuzhou Medical University (Grant XYFM202412).

## Disclosure

The authors take full responsibility for the final content of the publication. All authors have read and approved the final manuscript.

## Conflicts of Interest

The authors declare no conflicts of interest.

## Data Availability

The data that support the findings of this study are available from the corresponding author upon reasonable request.
